# A low-complexity region in human XRN1 directly recruits deadenylation and decapping factors in 5′–3′ messenger RNA decay

**DOI:** 10.1093/nar/gkz633

**Published:** 2019-07-24

**Authors:** Chung-Te Chang, Sowndarya Muthukumar, Ramona Weber, Yevgen Levdansky, Ying Chen, Dipankar Bhandari, Catia Igreja, Lara Wohlbold, Eugene Valkov, Elisa Izaurralde

**Affiliations:** 1 Department of Biochemistry, Max Planck Institute for Developmental Biology, Max-Planck-Ring 5, 72076 Tübingen, Germany; 2 Department of Molecular Biology, Max Planck Institute for Biophysical Chemistry, Am Fassberg 11, 37077 Göttingen, Germany

## Abstract

XRN1 is the major cytoplasmic exoribonuclease in eukaryotes, which degrades deadenylated and decapped mRNAs in the last step of the 5′–3′ mRNA decay pathway. Metazoan XRN1 interacts with decapping factors coupling the final stages of decay. Here, we reveal a direct interaction between XRN1 and the CCR4–NOT deadenylase complex mediated by a low-complexity region in XRN1, which we term the ‘C-terminal interacting region’ or CIR. The CIR represses reporter mRNA deadenylation in human cells when overexpressed and inhibits CCR4–NOT and isolated CAF1 deadenylase activity *in vitro*. Through complementation studies in an XRN1-null cell line, we dissect the specific contributions of XRN1 domains and regions toward decay of an mRNA reporter. We observe that XRN1 binding to the decapping activator EDC4 counteracts the dominant negative effect of CIR overexpression on decay. Another decapping activator PatL1 directly interacts with CIR and alleviates the CIR-mediated inhibition of CCR4–NOT activity *in vitro*. Ribosome profiling revealed that XRN1 loss impacts not only on mRNA levels but also on the translational efficiency of many cellular transcripts likely as a consequence of incomplete decay. Our findings reveal an additional layer of direct interactions in a tightly integrated network of factors mediating deadenylation, decapping and 5′–3′ exonucleolytic decay.

## INTRODUCTION

The spatial and temporal control of gene expression is crucial for many biological processes such as embryonic development, cell proliferation and immune response. One means to regulate gene expression is via control of mRNA levels through targeted mRNA decay. Cytoplasmic mRNA decay is usually initiated by shortening of the poly(A) tail, a process termed deadenylation (1–4), which is mediated by PAN2/PAN3 and CCR4–NOT deadenylase complexes (5–7). The CCR4–NOT is conserved in all eukaryotes and is principally responsible for the poly(A) tail shortening of the bulk transcriptome (4,8). The CCR4–NOT is a multiprotein complex, which comprises two active exonucleases—CCR4 and CAF1—as well as non-enzymatic protein subunits such as NOT2, NOT3 and CAF40, which are all assembled on a central NOT1 scaffold protein (9). The CCR4–NOT is recruited to transcripts by RNA-binding factors such as tristetraprolin (TTP), Nanos or Roquin, which bind specific sequences in the 3′ UTR of target mRNAs (10–18).

Following deadenylation, decay is either decapping-dependent in the 5′–3′ pathway or mRNAs are degraded via the exosome in 3′–5′ direction (6,19–21). Efficient removal of the protective 5′ cap structure from a transcript by the decapping enzyme DCP2 depends on a loose network of interacting factors such as DCP1, EDC3, EDC4, the DEAD-box helicase DDX6, LSm14A and the cytoplasmic LSm1–7/PatL1 protein complex (22–24). Decapping generates a 5′-monophosphorylated mRNA that is rapidly degraded by the conserved 5′–3′ exoribonuclease XRN1, which is the major cytoplasmic 5′–3′ exoribonuclease in eukaryotes (25). In addition, XRN1 mediates the degradation of endonucleolytically cleaved mRNA fragments generated by the nonsense-mediated decay (NMD) (26) or the siRNA-mediated gene silencing (27). XRN1 also functions in a buffering system that links mRNA production with degradation in yeast (28,29).

XRN1 co-localizes with decapping factors in processing (P-) bodies (30–32). In *Drosophila melanogaster*, XRN1 binds directly to the DCP1 decapping activator effectively coupling decapping with 5′–3′ exonucleolytic mRNA decay (33,34). Although the proline-rich DCP1-binding motif of XRN1 is not conserved in vertebrates, XRN1 binds a metazoan-specific factor EDC4 directly via a short linear motif in the XRN1 C-terminal region (termed EDC4-BM), thus maintaining direct interactions with the decapping network (33,35). In yeast and human cells, XRN1 also interacts with the decapping activator PatL1 (33,36–38), but it was not established whether human XRN1 and PatL1 interact directly. Human PatL1 maintains additional interactions with the CCR4–NOT, DDX6, DCP1 and DCP2 decapping factors (37,39), which functionally couples deadenylation and decapping (40). An emerging view of the 5′–3′ mRNA decay is that of a tightly coupled and a highly choreographed sequence of molecular events at the opposite ends of a transcript but many of the specific interactions and their functional significance remain poorly understood.

Here, we observed that the CCR4–NOT and XRN1 interact directly—a surprising finding given that exonucleolytic degradation must be preceded by poly(A) tail shortening and decapping in 5′–3′ mRNA decay. The interaction with CCR4–NOT is mediated by a low-complexity region C-terminal to the catalytic domain of XRN1, which we termed the ‘C-terminal interacting region’ or ‘CIR’. Overexpression of XRN1, as well as the CIR alone, represses deadenylation and subsequent decay of a reporter mRNA in human cells. The CIR also inhibits deadenylation catalyzed by either the intact CCR4–NOT or the isolated CAF1 deadenylase *in vitro*. The interaction of the CIR with the CCR4–NOT is mutually exclusive with the decapping activator PatL1. PatL1 also relieves the CIR-mediated inhibition of CAF1 deadenylation activity *in vitro*. At last, the interaction of the EDC4 decapping activator with XRN1 also counteracts the dominant negative effect of the CIR overexpression on mRNA decay. Collectively, we present findings that the XRN1 CIR serves an important and previously unappreciated functional role in the direct coupling of molecular events in the 5′–3′ mRNA decay pathway.

## MATERIALS AND METHODS

### DNA constructs

DNA constructs used in this study are listed in Supplementary Table S1. The plasmids for expression of the β-globin-6xMS2bs and the control β-globin-GAP (control) mRNAs were kindly provided by Dr J. Lykke-Andersen and were described previously (41). For recombinant production of the maltose-binding protein (MBP)-tagged human XRN1 CIR in *Escherichia coli*, the corresponding cDNA was inserted between the KpnI and BamHI restriction sites in the pETM-41P plasmid (EMBL, Heidelberg, Germany). A StrepII tag was inserted by site-directed mutagenesis at the C-terminal end. Human PNRC2 was amplified from cDNA and inserted between NcoI and BamHI restriction sites of the pnYC-pM plasmid (42), resulting in an MBP-tagged fusion construct cleavable by HRV-3C protease. A StrepII tag was inserted by site-directed mutagenesis at the C-terminal end.

### Co-immunoprecipitation assays and western blot analysis

For immunoprecipitation (IP) assays, HEK293T cells were seeded in 10 or 15 cm dishes and transfected with 30–50 μg total plasmid DNA using Lipofectamine 2000 (ThermoFisher) or calcium phosphate. The cells were washed 48 h after transfection with PBS and lysed in 1 ml NET buffer [50 mM Tris/HCl (pH 7.5), 150 mM NaCl, 1 mM ethylenediaminetetraacetic acid (EDTA), 0.1% (v/v) Triton-X-100, 10% (v/v) glycerol and supplemented with complete protease inhibitor cocktail (Sigma)]. IPs were performed as described previously (33). RNase A was added in all experiments. Antibodies used in this study are listed in Supplementary Table S2. All western blots were developed with the ECL western blotting analysis system (GE Healthcare) as recommended by the manufacturer.

### Pulldown assays

Full-length MBP-tagged PNRC2 was produced in *E. coli* BL21(DE3) Star cells (ThermoFisher) in LB medium at 30°C. For the purification, cells were lysed by sonication in a buffer containing 50 mM HEPES/NaOH (pH 7.0), 500 mM NaCl, 5% (v/v) glycerol and 2 mM dithiothreitol (DTT) supplemented with complete EDTA-free protease inhibitors (Sigma), 5 μg/ml DNase I and 1 mg/ml lysozyme. MBP/Strep-tagged PNRC2 was first isolated from the crude lysate on amylose resin (New England Biolabs) and eluted with lysis buffer supplemented with 25 mM D-(+)-maltose. The tags were left uncleaved. This was followed by size exclusion chromatography on a Superdex 75 16/60 column (GE Healthcare) equilibrated in the same buffer as used for lysis. The protein was then flash-frozen in liquid nitrogen for storage. Recombinant CCR4–NOT complex components were purified as previously described (17,43,44). The purification of the recombinant EVH1 domain of Dcp1 from fission yeast, as well as the recombinant PatL1-C, were also described previously (39,45). MBP/Strep-tagged CIR and MBP alone were produced at 20°C in *E. coli* BL21(DE3) Star cells. Cells were lysed by sonication in phosphate-buffered saline (PBS) with 0.1% (v/v) Tween-20. The cleared lysates or 30 μg of purified PNRC2 protein were incubated with 30 μl (50% slurry) of Strep-Tactin sepharose (IBA) resin for 1 h at 4°C. The resin was then washed twice with lysis buffer and once with binding buffer [50 mM Tris/HCl (pH 7.5), 150 mM NaCl]. Purified proteins were then added to the resin and incubated for 1 h at 4°C. After three washes with the binding buffer, bound proteins were eluted with the binding buffer supplemented with 2.5 mM biotin and analyzed by sodium dodecyl sulphate-polyacrylamide gel electrophoresis (SDS-PAGE) following Coomassie staining.

### Tethering assays

Tethering assays using the MS2 reporter system were performed as described previously (46). Briefly, HEK293T cells were cultured in six-well plates and transiently transfected with a mixture of four plasmids: 0.5 μg control plasmid (β-globin-GAP), 0.5 μg plasmid encoding the β-globin-6xMS2bs, 0.5 μg plasmids encoding the MS2-HA fusion protein (SMG7, Nanos1 or NOT1) and 2 μg plasmids encoding GFP-MBP, GFP-DCP2* (E148Q), GFP-POP2* (DExAA) or GFP-XRN1 (wild-type [WT] or fragments). The cells were harvested two days after transfection. Total RNA was isolated using the TriFast reagent (Peqlab) and analyzed by northern blot. To determine mRNA half-lives, transfected cells were treated with 10 μg/ml actinomycin D (final concentration) and harvested at the indicated time points. RNase H (New England Biolabs) digestion using a (dT)_15_ oligonucleotide was performed as recommended by the manufacturer.

### Deadenylation assays

Deadenylation assays were performed essentially as described previously (47). Indicated purified protein mixtures were incubated with 0.6 μM 5′-6-FAM- or ^32^P-labeled synthetic RNA substrate (5′-UCUAAAUA_20_–3′) at 37°C. Reactions were stopped by adding equal volumes of 2× RNA loading buffer [95% (v/v) formamide, 0.025% (w/v) SDS, 0.025% (w/v) bromophenol blue, 0.5 mM EDTA]. The reaction products were analyzed on a 20% polyacrylamide denaturing gel containing 7.0 M urea. The fluorescence was detected with a Typhoon imaging system (GE Healthcare). For experiments described and shown in Supplementary Figure S2B, HEK293T cells were transfected with a plasmid encoding GFP-CAF1 or GFP-CAF1*. Cells were lysed 48 h after transfection and GFP-tagged proteins were immunoprecipitated using a polyclonal rabbit anti-GFP antibody (made in house). The immunoprecipitates were incubated on ice with 12 μM purified MBP or MBP-tagged CIR for 10 min. Following two washes with NET buffer and one additional wash with the deadenylation buffer (47), the precipitates were examined by western blot or incubated at 37°C for the indicated time with the ^32^P-labeled RNA substrate. Reactions were then stopped and analyzed by denaturing PAGE followed by phosphorimaging using the Typhoon.

### Transcriptome sequencing (RNA-Seq) and ribosome profiling (Ribo-Seq)

HEK293T WT or XRN1-null cells were plated on 15 cm dishes 24 h before harvesting as previously described (48). Total RNA was extracted using the RNeasy Mini Kit (Qiagen) and a cDNA library was prepared using the TruSeq RNA Sample Prep Kit (Illumina). For Ribo-Seq, the original procedure (49) was followed with modifications described in (48). Two biological replicates were analyzed. RNA-Seq and Ribo-Seq libraries were sequenced using the HiSeq 3000 sequencing system (Illumina) using paired-end sequencing. During analysis, ribosomal RNA sequencing reads were filtered using Bowtie2 (50). Remaining reads were then mapped on the hg19 (UCSC) human genome with Tophat2 (51). Ribosome profiling reads were analyzed for three-nucleotide periodicity using the RiboTaper program to identify actively translating ribosomes (48). Reads corresponding to the lengths of 29 and 30 nt were selected because they showed the most significant three-nucleotide periodicity and were then used for mapping on the human genome with Tophat2. For RNA-Seq, 17.6–19.9 million reads (89.4–90.0%) of input reads were mapped. For Ribo-Seq, 2.5–5.1 million reads (90.8–95.6%) of input reads were mapped. Read count analysis was done with an R/Bioconductor package QuasR (52). A threshold of ‘fragments per kilobase of transcript per million mapped reads’ (FPKM) >2 was applied to select genes for subsequent differential gene expression analysis with an R/Bioconductor package edgeR (53,54). Translational efficiency (TE) was estimated using the statistical framework and analysis as implemented in the RiboDiff program (55). For Supplementary Figure S4, individual transcript RNA sequencing tracks were visualized using the Integrative Genomics Viewer visualization tool (56,57).

### 5′–3′ exoribonuclease assay

The 5′–3′ exoribonuclease assay was described previously (58). Following the lysis of HEK293T cells overexpressing GFP-tagged MBP or XRN1 catalytic domain (residues 1–1173), tagged proteins were precipitated using anti-GFP antibody (made in house). The immunoprecipitates were tested for exonuclease activity using ^32^P 5′-labeled synthetic RNA (5′-UCUAAAUA_20_–3′). Reactions were carried out at 37°C for indicated time in a buffer containing 30 mM Tris/HCl (pH 8.0), 2 mM MgCl_2_, 50 mM NH_4_Cl, 0.5 mM DTT, 20 mg/ml acetylated bovine serum albumin (BSA). Reactions were stopped by adding 2× RNA loading buffer. The reaction products were analyzed by denaturing urea-PAGE. The radioactivity was detected and quantified with a Typhoon imaging system (GE Healthcare).

### Electrophoretic mobility shift assay (EMSA)

Binding reactions contained 200 nM (final concentration) of fluorescently labeled RNA (the same as used in deadenylation assays) and 1–2 μM (final concentration) of the indicated proteins in 10 μl total reaction volume of binding buffer [20 mM Tris/HCl (7.5), 10 mM NaCl, 2 mM MgCl_2_, 0.1% (w/v) BSA, 0.1% (w/v) Orange G, 3% (w/v) Ficoll 400]. For competition assays with PatL1-C, the concentration of CIR was kept constant at 2 μM and PatL1-C was added at 1:1 and 1:2 molar ratio. The highest PatL1-C concentration (4 μM) was used as a control. The RNA-protein complexes were analyzed by electrophoresis on a 10% nondenaturing polyacrylamide gel in Tris-borate-EDTA buffer (pH 8.3) at 10 V/cm.

### CRISPR/Cas9-mediated gene editing

The HEK293T XRN1-null cell line was generated by CRISPR/Cas9-mediated gene editing as described previously (59). The guide RNA targeting exon 6 of the human *XRN1* gene (5′-AGAGAAGAAGTTCGATTTGG-3′) was designed using the ATUM CRISPR gRNA Design tool. The absence of detectable XRN1 protein was confirmed by western blotting. Sanger sequencing of the targeted locus confirmed the presence of a 17 bp deletion within the targeted exon resulting in a frameshift. Subsequent RNA-Seq analysis revealed the retention of intron 5 in all detectable *XRN1* transcripts consistent with a disruption of the splice acceptor site by the deletion (Supplementary Figure S4A).

### 5′-phosphate-dependent exonuclease assay

The integrity of the 7-methylguanosine cap structure on the 5′ ends of transcripts was verified with a 5′-phosphate-dependent exonuclease assay. A total of 10 μg RNA extracted from the indicated tethering assays was incubated in a 20 μl total reaction volume with 1 unit of Terminator 5′-phosphate-dependent exonuclease (Epicentre) for 60 min at 30°C. The Terminator was omitted in the control. The reaction was then stopped by addition of phenol followed by standard extraction and ethanol precipitation. RNA levels were visualized by northern blotting.

## RESULTS

### XRN1 directly interacts with CCR4–NOT via multiple binding sites

**Figure 1.**
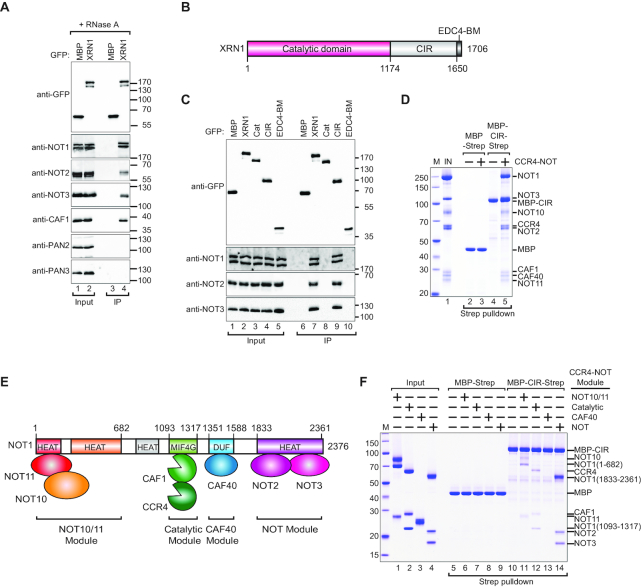
XRN1 interacts directly with CCR4–NOT via a low-complexity region. (**A**) The interaction of GFP-tagged XRN1 with endogenous deadenylation factors. The proteins were immunoprecipitated using anti-GFP antibodies. GFP-MBP served as a negative control. Inputs (10%) and bound fractions (IP; 30%) were analyzed by western blotting using the indicated antibodies. The size markers (kDa) are shown on the right of the panel. (**B**) The domain organization of human XRN1. XRN1 consists of a catalytic domain (indicated in red) and a C-terminal variable, low-complexity region comprising the C-terminal interacting region (CIR, light gray) and the EDC4-binding motif (EDC4-BM, dark gray). The residue numbers are indicated at the domain boundaries. (**C**) The interaction of XRN1 (full length or the indicated fragments) with the endogenous CCR4–NOT subunits. The proteins were immunoprecipitated using anti-GFP antibodies and analysed as described in (A). (**D**) A streptavidin pulldown assay showing the interaction of Strep-tagged CIR with the recombinant purified CCR4–NOT. Strep-tagged MBP served as a negative control. The size markers (kDa) denoted by ‘M’ are shown on the left of the panel. (**E**) Schematic representation of the CCR4–NOT complex and subcomplex modules. (**F**) A streptavidin pulldown assay showing the interaction of Strep-tagged CIR with recombinant purified CCR4–NOT modules. Strep-tagged MBP served as a negative control.

XRN1 interacts directly with decapping factors in human and *Drosophila melanogaster (Dm)* cells. To test if XRN1 can bind other decay factors, we probed interactions with CCR4–NOT and PAN2/PAN3 deadenylase complexes in co-IP assays. Exogenously expressed GFP-XRN1 immunoprecipitated all tested endogenous subunits of the CCR4–NOT including NOT1, NOT2, NOT3 and CAF1 in the presence of RNase A suggesting that these interactions were not bridged by RNA (Figure 1A). In contrast, GFP-XRN1 did not immunoprecipitate endogenous PAN2 or PAN3 (Figure 1A).

To delineate the region of XRN1 responsible for the interaction with CCR4–NOT, we performed co-IP assays with the N-terminal catalytic domain (residues 1–1173), the C-terminal low-complexity region (residues 1174–1649) and the EDC4-binding motif (residues 1650–1706; EDC4-BM) (Figure 1B). The XRN1 C-terminal low-complexity region, but not the other fragments, precipitated the endogenous NOT1, NOT2 and NOT3 (Figure 1C) and we termed this region the ‘C-terminal interacting region’ or ‘CIR’.

We then asked if the interaction with CCR4–NOT is direct. The MBP- and streptavidin (Strep)-tagged recombinant CIR directly binds the reconstituted recombinant, complete CCR4–NOT complex in a pulldown assay (Figure 1D, lane 5). To identify which components of the CCR4–NOT mediate binding to the CIR, we then examined the MBP/Strep-CIR interaction with individually reconstituted CCR4–NOT subcomplexes in a pulldown. Tested CCR4–NOT components consisted of different segments of NOT1 in complex with its respective interaction partners: the N-terminal NOT1/10/11 module, the catalytic module (NOT1/CAF1/CCR4a), the CAF40 module (NOT1/CAF40) and the C-terminal NOT module (NOT1/2/3) (Figure 1E). The CIR interacted weakly with the NOT1/10/11 subcomplex as well as the catalytic module (Figure 1F, lanes 11 and 12) and no binding was detected with the CAF40 module under the pulldown conditions (Figure 1F, lane 13). A stable interaction was observed between the CIR and the NOT module (Figure 1F, lane 14). To verify the stringency of the pulldown assay, we tested MBP-Strep tag alone as well as an MBP/Strep-tagged PNRC2 protein, which is a low-complexity protein with some similarity in its sequence properties to the XRN1 CIR. Neither detectably interacted with CCR4–NOT components but PNRC2 pulled down the EVH1 domain of Dcp1 from fission yeast as a positive control (Figure 1F, lanes 6–9 and Supplementary Figure S1A).

### XRN1 overexpression inhibits deadenylation in human cells

**Figure 2.**
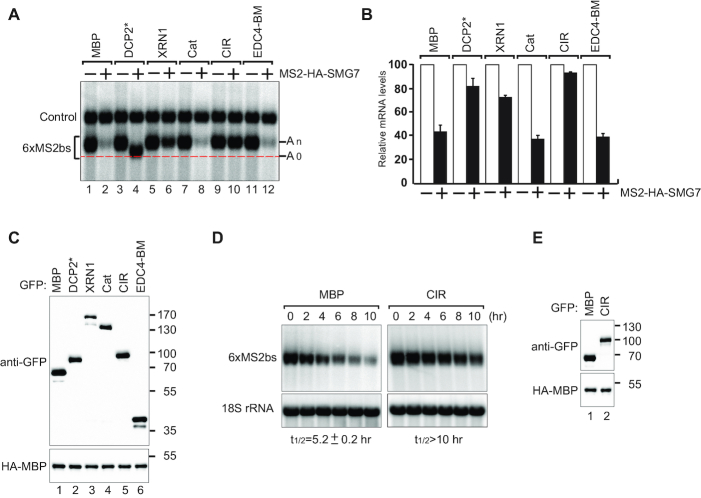
XRN1 overexpression inhibits mRNA degradation. (**A**) Human HEK293T cells were transfected with a mixture of four plasmids: one expressing β-globin-6xMS2bs mRNA, another expressing MS2-tagged HA (−) or HA-SMG7 (+), a third expressing a transfection control containing the β-globin gene fused to the GAPDH fragment (41) but lacking MS2 binding sites (control), and a fourth expressing either GFP-tagged MBP, DCP2* (E148Q mutant) or XRN1 (full length or the indicated fragments). A northern blot of representative RNA samples is shown. The positions of the polyadenylated (A_n_) and the deadenylated (A_0_) forms of the β-globin-6xMS2bs reporter are indicated on the right. A red dotted line additionally marks the fast migrating deadenylated (A_0_) form. (**B**) The β-globin-6xMS2bs mRNA levels were normalized to those of the control mRNA. These normalized values were set to 100 in cells expressing MS2-HA (white bars). The mean values for relative mRNA levels in cells expressing MS2-HA-SMG7 were estimated with standard deviations (SD) from three independent experiments (black bars). (**C**) A western blot demonstrating equivalent expression of the GFP-tagged proteins. HA-MBP served as a transfection control. (**D**) Representative northern blots showing the decay of β-globin-6xMS2bs mRNA tethered to MS2-HA-SMG7 in the presence of either overexpressed GFP-MBP or GFP-CIR. mRNA levels were analyzed following transcriptional shutoff with actinomycin D treatment. β-globin-6xMS2bs mRNA levels were normalized to 18S rRNA levels and quantified as a function of time. The mRNA half-life (t_1/2_) ± SD indicated below each panel was calculated by fitting an exponential decay function curve to data obtained from three independent experiments. (**E**) A western blot demonstrating the equivalent expression of the GFP-tagged proteins.

To probe the functional link between XRN1 and CCR4–NOT, we examined the degradation of a reporter mRNA in human HEK293T cells overexpressing GFP-tagged XRN1 (Figure 2A–C). Overexpression of XRN1 levels in *Dm* cells was demonstrated to block decapping in a dominant negative manner indicating that this approach can provide insights into the processes regulated by XRN1 (33). We tethered the NMD factor SMG7 to a β-globin mRNA reporter containing six MS2 binding sites in the 3′ UTR (β-globin-6xMS2bs mRNA) (41). SMG7 promotes deadenylation, decapping and subsequent 5′–3′ exonucleolytic degradation of the reporter mRNA (35,46). As expected, the expression of MS2-HA-SMG7 elicited degradation of the β-globin-6xMS2bs mRNA but did not affect the expression of the control mRNA lacking the MS2 binding sites (Figure 2A, lanes 1, 2 and B). Overexpression of a catalytically inactive mutant of the decapping enzyme DCP2* (E148Q) (60) inhibited decapping and a stabilized deadenylated (A_0_) mRNA reporter was readily detected as a band with increased mobility compared to a polyadenylated (A_n_) mRNA (Figure 2A, lane 4 and B). Unexpectedly, we observed an accumulation of the slow-migrating and polyadenylated (A_n_) form of the reporter in cells overexpressing XRN1 (Figure 2A, lane 6 and B). This suggested that the observed defect in SMG7-induced reporter mRNA decay was a consequence of the repression of deadenylation.

We then overexpressed GFP-tagged XRN1 fragments in HEK293T cells and performed tethering assays. Although all fragments were expressed at comparable levels (Figure 2C), the overexpression of the XRN1 CIR region, but not the catalytic domain of XRN1 or the EDC4-BM, elicited an almost complete block of SMG7-mediated mRNA decay as evident by the accumulation of polyadenylated (A_n_) reporter (Figure 2A, lane 10 and B). We then asked if the observed inhibitory effect of CIR on deadenylation was specific to SMG7. To test this, we tethered either NOT1 or the CCR4–NOT recruitment factor Nanos1 to induce deadenylation and 5′–3′ mRNA decay (15,16). In both cases, we observed that overexpression of exogenous CIR resulted in strong repression of mRNA decay (Supplementary Figure S1B).

To assess the extent of polyadenylation of the reporter accumulating in cells expressing the GFP-CIR, we performed an oligo(dT)-directed ribonuclease H (RNase H) cleavage assay. The RNase H treatment of mRNA isolated from cells expressing the GFP-CIR led to a shift in the migration of the β-globin-6xMS2bs reporter to match that of the deadenylated (A_0_) reporter (Supplementary Figure S1C, lanes 6, 8 versus lanes 2, 4), consistent with the notion that the β-globin-6xMS2bs mRNA was polyadenylated. We then asked if the reporter was decapped. To test this, we treated the mRNA with the Terminator 5′-phosphate exonuclease. The transcripts were resistant to this treatment, which is consistent with the presence of an intact 5′ cap (Supplementary Figure S1D). Collectively, this evidence suggests that the CIR-mediated block of reporter mRNA decay is most likely the consequence of the repression of deadenylation rather than decapping and/or subsequent 5′–3′ exonucleolytic activity.

The mRNA decay rates are coupled to polymerase II-mediated transcription in both yeast and mammalian cells (28,29,61–63). Interestingly, in yeast, XRN1 has a key role in the buffering system linking mRNA synthesis with decay (28,29). We asked whether the observed increase in reporter mRNA levels correlated with the XRN1 CIR overexpression is indeed the consequence of a defect in mRNA decay rather than increased transcription. To test this, we analyzed the β-globin-6xMS2bs reporter mRNA levels in a time course following transcriptional shut-off via an actinomycin D treatment. In control cells, tethering of MS2-HA-SMG7 resulted in a half-life of the β-globin-6xMS2bs mRNA of ∼5 h (Figure 2D and E). In contrast, CIR overexpression extended the half-life to over 10 h (Figure 2D and E).

### The XRN1 CIR inhibits CAF1 deadenylation activity *in vitro*

**Figure 3.**
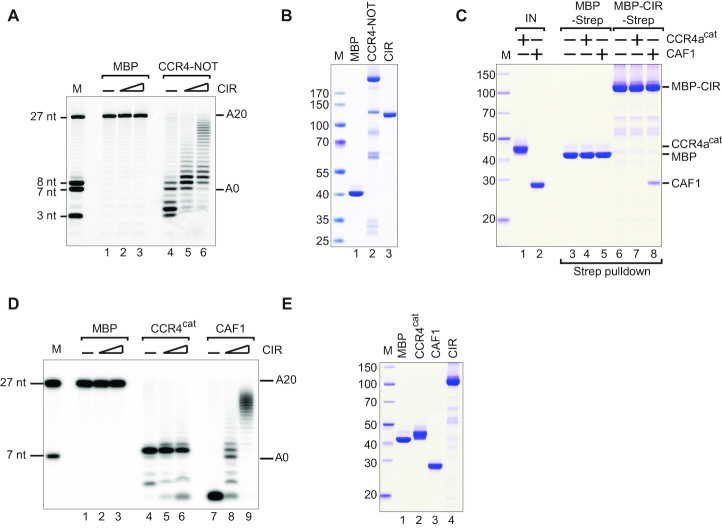
The CIR inhibits CAF1-mediated deadenylation *in vitro*. (**A**) A total of 100 nM purified CCR4–NOT complex or MBP alone was incubated at 37°C for 60 min with a 27 nt 5′-6-FAM-labeled RNA substrate containing a homopolymeric stretch of 20 As at the 3′ end. The purified recombinant CIR was titrated as a series of 0, 6 and 12 μM concentrations as indicated. (**B**) Coomassie-stained SDS-PAGE analysis of the recombinant proteins used in the deadenylation assay. (**C**) A streptavidin pulldown assay showing the interaction of Strep-tagged CIR with 10 μM purified CAF1 or the catalytic domain of CCR4a (CCR4a^cat^, residues 159–557). Strep-tagged MBP served as a negative control. ‘IN’ denotes input. The size markers (kDa) denoted by ‘M’ are shown on the left of the panel. (**D**) Purified CCR4^cat^, CAF1 or MBP alone (0.6 μM) was incubated with the RNA substrate at 37°C for 60 min. The purified recombinant CIR was titrated as a series of 0, 6 and 12 μM concentrations as indicated. (**E**) Coomassie-stained SDS-PAGE analysis of the recombinant proteins used in the (D).

To test whether the XRN1 CIR can directly inhibit the CCR4–NOT catalytic activity, we performed *in vitro* deadenylation assays using purified recombinant CCR4–NOT. As a substrate, we used a 27 nucleotide (nt) RNA substrate labeled with fluorescein at the 5′ end for detection and containing a 20 nt poly(A) at the 3′ end (Figure 3A and B). The CCR4–NOT completely degraded the polyadenosine sequence within 60 min and was even active toward the non-poly(A) sequence (Figure 3A, lane 4). We then titrated recombinant MBP/Strep-tagged CIR in this assay and observed partial inhibition of the CCR4–NOT deadenylase activity in a dose-dependent manner (Figure 3A, lanes 5, 6 versus lane 4).

The two enzymatic components of the CCR4–NOT—CAF1, a DEDD type ribonuclease, and CCR4, an EEP domain-containing nuclease (64–68)—are both required for deadenylation in human cells (69). We asked whether CIR interacts directly with either CAF1 or CCR4a deadenylase. We observed that MBP/Strep-tagged CIR binds to the recombinant CAF1 but not the recombinant catalytic domain of CCR4a (CCR4a^cat^) in a pulldown assay (Figure 3C, lane 8 versus lane 7). We then assayed the deadenylation activity of full-length CAF1 and observed that this enzyme was highly active but not specific for polyadenosine (Figure 3D, E). Interestingly, when we titrated the MBP/Strep-tagged CIR in the deadenylation assays, we observed that the CIR strongly inhibited CAF1 but not CCR4a^cat^-mediated deadenylation (Figure 3D, lanes 8, 9 versus 5, 6).

We then asked if the recombinant XRN1 CIR can also inhibit CAF1 isolated from human cells, rather than the recombinant enzyme. We immunoprecipitated overexpressed GFP-tagged CAF1 from HEK293T cells, added the recombinant CIR or the MBP control and then extensively washed the precipitate. Expression of an inactive CAF1* catalytic mutant (DExAA) served as a control. Exogenously expressed GFP-CAF1 and -CAF1* were incorporated into the endogenous CCR4–NOT as they efficiently precipitated endogenous NOT1, NOT2, NOT3 and CCR4a as evident from the western blot (Supplementary Figure S2A). Importantly, in the presence of the CIR, CAF1 did not dissociate from the CCR4–NOT (Supplementary Figure S2A, lane 4). The GFP-CAF1 immunoprecipitate, but not the GFP-CAF1* and GFP-MBP controls, readily deadenylated the 27 nt RNA substrate (Supplementary Figure S2B, lanes 5–8). Addition of the purified recombinant CIR, however, significantly reduced the deadenylation activity of GFP-CAF1 immunoprecipitate in a time course assay (Supplementary Figure S2B, lanes 13–16).

### PatL1 competes with the CCR4–NOT for direct binding to the XRN1 CIR

**Figure 4.**
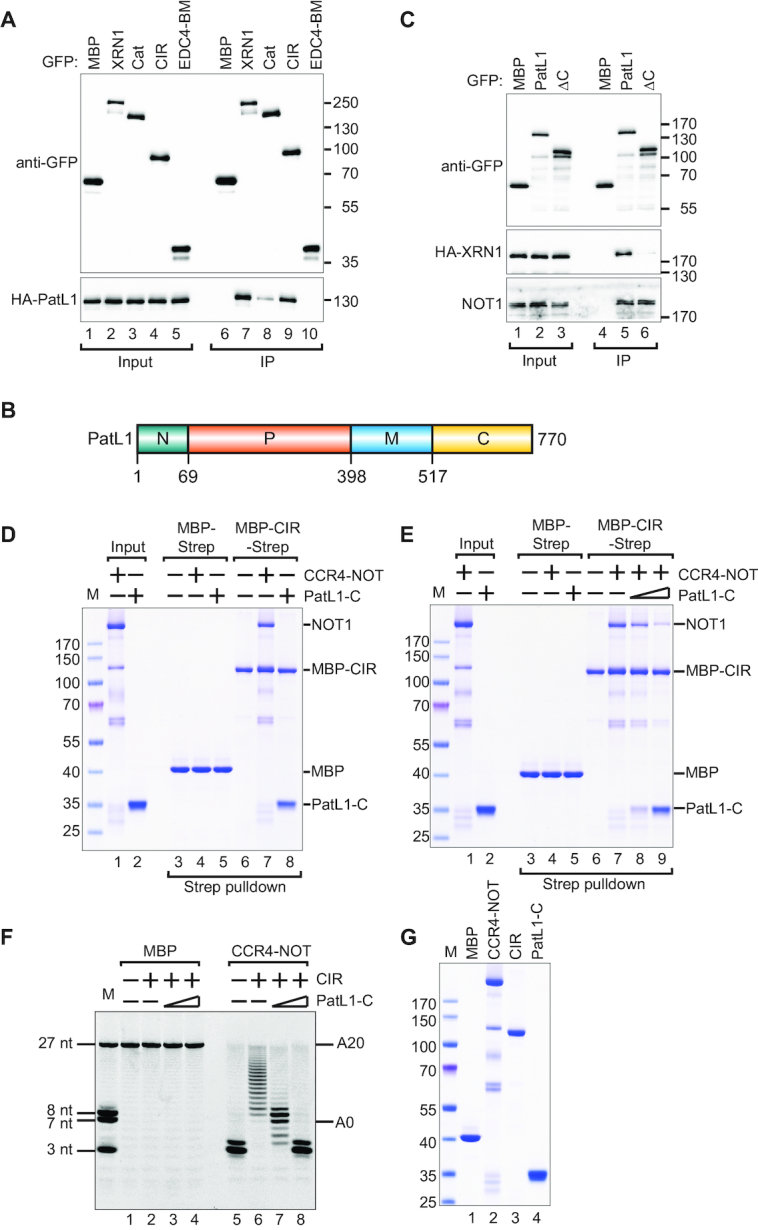
PatL1 competes with the CCR4–NOT for binding to the CIR. (**A**) The interaction of GFP-tagged XRN1 (full-length or fragments) with HA-tagged PatL1 in HEK293T cell lysates. The proteins were immunoprecipitated using anti-GFP antibodies. GFP-MBP served as a negative control. Inputs (10% for GFP-tagged proteins and 20% for HA-tagged proteins) and bound fractions (IP; 30%) were analyzed by western blotting using the indicated antibodies. The size markers (kDa) are shown on the right of the panel. (**B**) The domain organization of human PatL1. PatL1 consists of a conserved N-terminal sequence (N, green), the P-rich region (P, orange), the Mid domain (M, blue) and the C-terminal domain (C, yellow). The residue numbers are indicated at the domain boundaries. (**C**) The interaction between GFP-PatL1 (full-length or ΔC) and HA-tagged XRN1 in HEK293T cells. The proteins were immunoprecipitated using anti-GFP antibodies. GFP-MBP served as a negative control. Inputs (10%) and bound fractions (IP; 30%) were analyzed by western blotting using the indicated antibodies. (**D**) A streptavidin pulldown assay showing the interaction of Strep-tagged CIR with purified recombinant CCR4–NOT complex (0.5 μM) or purified recombinant PatL1-C (3.5 μM). Strep-tagged MBP served as a negative control. The size markers (kDa) denoted by ‘M’ are shown on the left of the panel. (**E**) A streptavidin pulldown showing the interaction of purified recombinant MBP/Strep-tagged CIR with purified recombinant CCR4–NOT complex (0.5 μM) when challenged with a titration of purified recombinant PatL1-C as a series of 0, 1.75 and 3.5 μM concentrations as indicated. (**F**) A total of 100 nM purified CCR4–NOT complex or MBP alone was incubated at 37°C for 60 min with the RNA substrate and 12 μM purified recombinant CIR. The purified recombinant PatL1-C was titrated as a series of 0, 8.5 and 34 μM concentrations as indicated. (**G**) Coomassie-stained SDS-PAGE analysis of the recombinant proteins used in the deadenylation assay.

The human decapping activator PatL1 immunoprecipitates with XRN1 and the interaction between them was mapped to a region comprising both the catalytic domain and the CIR (Figure 1B) (33). Using co-IP, we observed that the catalytic domain is redundant for this interaction and that the CIR is necessary and sufficient for XRN1 to interact with PatL1 (Figure 4A).

To delineate more precisely the XRN1-binding region on PatL1, we performed co-IP of XRN1 with either full-length PatL1, N+P (residues 1–398) or M+C (residues 399–770) fragments (Figure 4B). This revealed that XRN1 interacts with the PatL1 M+C region, but not with the N+P region (Supplementary Figure S3A). The PatL1 ΔC truncated construct, which lacks the C-terminal portion (residues 517–770) (Figure 4B), does not precipitate XRN1 suggesting that this region of PatL1 is required to bind XRN1. However, the PatL1 ΔC fragment retains binding to NOT1 (Figure 4C, lane 6). The important implication here is that PatL1 interactions with XRN1 and CCR4–NOT employ different regions and are therefore unlikely to be mutually exclusive. In pulldown assays with recombinant proteins, we observed that the PatL1 C-terminal region, which we termed PatL1-C, is necessary and sufficient for the direct interaction with the XRN1 CIR (Figure 4D, lane 8).

We then asked if PatL1-C and the CCR4–NOT compete for binding to XRN1. To test this, we titrated recombinant PatL1-C into the reconstituted recombinant CIR/CCR4–NOT complex. Strikingly, PatL1-C efficiently displaced the CCR4–NOT complex from the CIR in a dose-dependent manner (Figure 4E, lane 7–9), suggesting that CCR4–NOT and PatL1 interactions with XRN1 are mutually exclusive.

Next, we asked if PatL1 can interfere with the CIR-mediated inhibition of CAF1 deadenylase activity. We then titrated increasing concentrations of the recombinant PatL1-C in deadenylation assays with recombinant MBP/Strep-tagged CIR and the CCR4–NOT (Figure 4F and G). Strikingly, the titration of recombinant PatL1-C relieved the CIR-mediated inhibition of the CCR4–NOT deadenylase activity (Figure 4F, lanes 6 versus 7, 8).

Interestingly, we also observed that CIR binds a synthetic RNA in an electrophoretic mobility shift assay much more efficiently than the isolated CAF1 and CCR4a nucleases (Supplementary Figure S3B). This indicated that the CIR may exert its inhibitory effect by effectively competing for and sequestering the RNA substrate in addition to direct interactions with the deadenylase. However, the addition of PatL1-C did not affect the interaction of the CIR with RNA while relieving the CIR-mediated inhibition of deadenylation (Supplementary Figure S3C). This suggests that CIR-mediated deadenylation inhibition is unlikely to be the sole consequence of direct interactions with the RNA.

### Loss of XRN1 leads to accumulation of mRNA decay intermediates

To investigate the function of the XRN1 CIR in 5′–3′ mRNA decay, we generated an XRN1-null HEK293T cell line by CRISPR/Cas9-mediated gene editing. We first verified the targeting of the genomic locus by sequencing (‘Materials and Methods’ section) and then confirmed the absence of XRN1 by western blot (Supplementary Figure S5A). The XRN1-null cells were viable and proliferated at normal rates consistent with previous reports (70).

**Figure 5.**
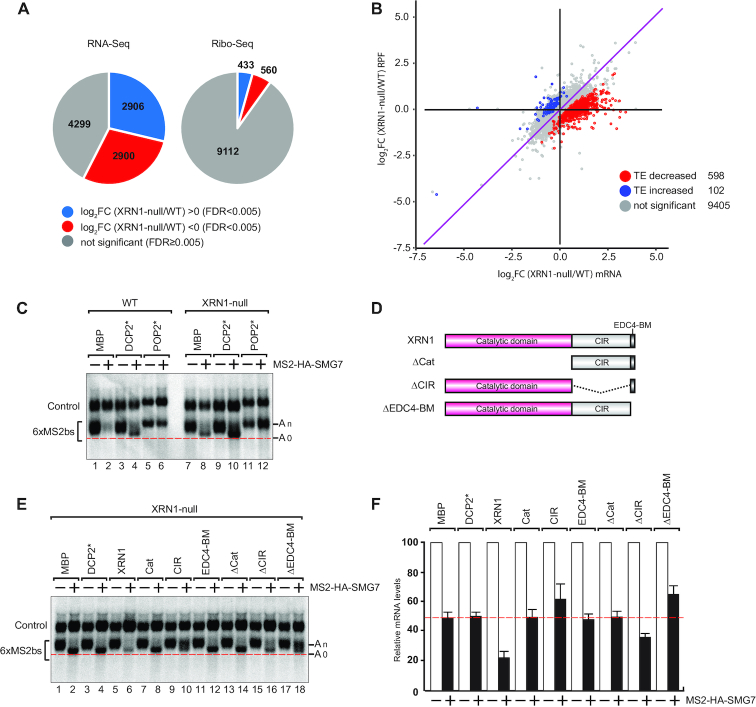
XRN1 function in 5′–3′ reporter mRNA decay. (**A**) Pie charts indicating the fractions and absolute numbers of differentially expressed genes derived from the analysis of the transcriptome and the translatome of HEK293T WT and XRN1-null cells by RNA-Seq and Ribo-Seq, respectively. Two biological replicates of each cell line were analyzed. The RNA-Seq analysis indicated that 4299 (gray) of the total 10 105 genes selected using FPKM > 2 cutoff showed no significant differences between the two cell lines (FDR ≥ 0.005). 2906 genes were significantly upregulated (blue) while 2900 genes were downregulated (red) using a fold change >0 on log2 scale with an FDR < 0.005 to determine abundance. Significantly fewer genes showed differences between the WT and the XRN1-null cells at the level of translation with 433 genes showing an increase in abundance of ribosomal footprints (blue) while 560 genes were decreased (red) as indicated by the Ribo-Seq analysis. (**B**) Comparative analysis of TE in WT and XRN1-null cells on a genome-wide scale as a ratio of ribosomal occupancy to mRNA abundance. Logarithmic change in mRNA abundance (log_2_FC) on the horizontal axis is plotted as a scattergraph against the log_2_FC of the ribosome-protected fragments (RPF). Each colored dot represents an individual gene. Correlation between changes in mRNA levels with the ribosomal occupancy is evident by a general trend along a diagonal indicated by a purple line. Correlation outliers that do not follow this trend are identified using a statistical test based on dispersion estimates as implemented in RiboDiff. These outliers are characterized as having either an increase in TE (102 genes, blue) or a decrease (598 genes, red) with most of the genes showing no evidence of change in the TE. (**C**) Tethering assay results in human HEK293T WT or XRN1-null cells, essentially performed as in Figure 2A. The transfection mixture included plasmids expressing either GFP-tagged MBP, DCP2* (E148Q) or POP2* (DExAA). (**D**) Schematic overview of the XRN1 deletion constructs. (**E**) Complementation assays with XRN1 deletion constructs in HEK293T XRN1-null cells performed essentially as in Figure 2A. The transfection mixture included plasmids expressing the indicated GFP-tagged constructs. (**F**) The β-globin-6xMS2bs mRNA levels were normalized to those of the control mRNA. These normalized values were set to 100 in cells expressing MS2-HA (white bars). The mean values for relative mRNA levels in cells expressing MS2-HA-SMG7 were estimated with standard deviations (SD) from three independent experiments (black bars). The red dotted line denotes the residual reporter mRNA level detected in HEK293T XRN1-null cells on SMG7 tethering.

We then wondered how the absence of XRN1 would affect the transcriptome and the translatome in cells. To this end, we sequenced the mRNA pool (RNA-Seq) and performed ribosome profiling (Ribo-Seq) (49) of the XRN1-null HEK293T cells and compared with that of the WT cells (Figure 5A and B). Differential gene expression analysis revealed that 2906 mRNAs were significantly upregulated in the XRN1-null cell line (Figure 5A). We then analyzed changes in the TE by comparing the Ribo-Seq and RNA-Seq datasets (Figure 5B) (55,71). The TE was significantly reduced for 598 transcripts in the XRN1-null cells but just 102 transcripts were evidently enhanced in their TE. Importantly, significant upregulation in mRNA levels was negatively correlated with ribosomal occupancy in the absence of XRN1 for a range of cellular transcripts resulting in their decreased TE (Figure 5B).

A closer inspection of read data coverage in XRN1-regulated target mRNAs with decreased TE, such as *DDX11* and *WASH3p*, yields some clues for the loss of ribosomal footprints despite upregulation of transcript levels. In these transcripts we observed a strong bias in the distribution of the RNA-Seq reads mapped across all exons. Specifically, in XRN1-null cells there is a localized and sudden increase in the number of mapped reads toward the 3′ end (Supplementary Figure S4 A–C). This observation may be explained by an accumulation of fragmented or cleaved mRNAs in XRN1-null cells – possibly as a result of NMD. Endonucleolytic cleavage generates both, 5′ and 3′ mRNA fragments, but the 5′ fragments are not observed by RNA-Seq because they lack poly(A) tails. Thus, we have preferentially selected and sequenced uncapped 3′ mRNA fragments that accumulate but are not translated in XRN1-null cells. Whether such endonucleolytic cleavage indeed occurs in HEK293T cells—and can be observed in the XRN1-null background—remains to be verified but this data is consistent with a pervasive accumulation of mRNA decay intermediates, which are not competent for cap-mediated translation.

### EDC4 binding to XRN1 relieves CIR-mediated deadenylation repression

In XRN1-null cells, SMG7-induced mRNA degradation of the β-globin-6xMS2bs reporter was impaired but not completely repressed. However, tethering of MS2-HA-SMG7 in XRN1-null, but not in WT cells, led to the accumulation of the fast migrating deadenylated (A_0_) and decapped reporter (Figure 5C, lanes 2, 8 and Supplementary Figure S5B) consistent with impairment of the 5′–3′ exonucleolytic degradation in the absence of XRN1. The overexpression of DCP2* (E148Q) stabilized the deadenylated mRNA decay intermediate, which was capped in WT and XRN1-null cells (Figure 5C, lanes 4, 10 and Supplementary Figure S5B). The overexpression of the catalytic inactive mutant paralog of CAF1, the deadenylase POP2* (DExAA) (15), stabilized the polyadenylated reporter in both cell lines (Figure 5C, lanes 6, 12). Taken together, this data indicates that deadenylation and decapping are not blocked in the absence of XRN1 in 5′–3′ decay in human cells consistent with previous observations in HeLa and *Dm* Schneider 2 (S2) cells (33,72).

We then performed tethering assays using MS2-tagged SMG7 and the β-globin-6xMS2bs reporter as above and complemented the cells with either GFP-tagged full-length XRN1 or fragments (Figure 5D-F and Supplementary Figure S5C). Exogenous expression of full-length XRN1 restored the exonucleolytic decay of the reporter in XRN1-null cells as the deadenylated decay intermediate (A_0_) no longer accumulated (Figure 5E, lane 2 versus 6). The XRN1 catalytic domain alone was not sufficient to restore exonucleolytic activity (Figure 5E, lane 6 versus 8), and we verified the catalytic activity of this domain in an *in vitro* 5′–3′ exoribonuclease assay (Supplementary Figure S5D) (58). However, the catalytic domain is required as the XRN1 fragment in which this domain was deleted was inactive (Figure 5E, lane 14). The C-terminal EDC4-BM alone also failed to restore XRN1 activity (Figure 5E, lane 12), but the CIR overexpression correlated with the appearance of partially deadenylated mRNA decay intermediates in XRN1-null cells (Figure 5E, lane 10). This suggests that the CIR exerts a dominant negative effect on deadenylation and/or decapping prior to 5′–3′ exonucleolytic decay (Figure 5E, lane 10).

Intriguingly, overexpression of the fragment comprising the CIR and the EDC4-BM (ΔCat) did not reproduce the repression of decay observed with the CIR alone in the XRN1-null background, which suggests that interactions with EDC4 counteract the dominant negative effect of the CIR on mRNA decay (Figure 5E, lanes 10, 14). The importance of interactions with EDC4 is further underscored by the observation that removing just the EDC4-BM from XRN1 also failed to complement and restore decay in XRN1-null cells (Figure 5E, lane 18 and F), which is consistent with previous observations in *Dm* S2 cells (33). Another interesting observation is that abrogating the interaction of EDC4 with XRN1 also stabilized the polyadenylated reporter similar to what was observed when the CIR was overexpressed (Figure 5E, lane 18). At last, the XRN1 fragment lacking the CIR did not fully complement SMG7-induced 5′–3′ decay consistent with the CIR maintaining interactions important for the XRN1 function in decay (Figure 5E, lane 16 and F).

**Figure 6.**
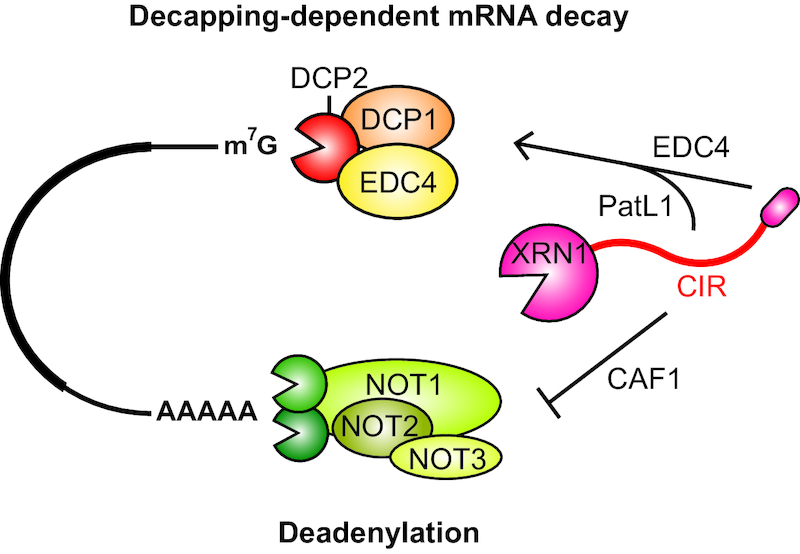
A summary of interactions mediated by XRN1 in 5′–3′ mRNA decay. XRN1 directly interacts with the decapping activator EDC4 via the short linear motif in its very C-terminal end (EDC4-BM, red oval) providing a direct mechanistic link between decapping and subsequent exonucleolytic mRNA decay. XRN1 can also directly interact with the CCR4–NOT deadenylase complex via the low-complexity ‘C-terminal interacting region’ (CIR) and block CAF1-dependent mRNA deadenylation. PatL1 competes with the CCR4–NOT for binding to the CIR and relieves CIR-mediated inhibition of CCR4–NOT catalytic activity *in vitro*.

Taken together, although the complementation studies point to an overall stimulatory role of the XRN1 CIR in decapping-dependent mRNA decay, it is presently not clear, however, whether the observed dominant negative effect of the XRN1 CIR on deadenylation can be attributed solely to the CIR or is a consequence of failure to bind EDC4, or it is indeed a combined effect. The complementation analysis revealed, however, that all regions of XRN1 are required for its function in 5′–3′ decay: the catalytic domain contains the 5′–3′ exonuclease activity; the EDC4-BM bridges interactions with the decapping machinery; and the CIR contributes either by interaction with the CCR4–NOT and/or PatL1 (Figure 6).

## DISCUSSION

XRN1 is a highly processive 5′–3′ exoribonuclease with a principal function in the degradation of the products of deadenylation-dependent decapping. In addition, XRN1 also interacts with distinct decapping factors in various species suggesting a conserved upstream regulatory role in decapping-dependent mRNA decay (33,34,36,38). In this study, we show that human XRN1 interacts with the CCR4–NOT deadenylation complex as well as a decapping factor PatL1, thus extending the repertoire of XRN1 interactors beyond the previously reported association with the decapping factor EDC4. XRN1 appears to repress deadenylation when overexpressed in human cells and we have mapped this repressive function to the low-complexity C-terminal region of XRN1, which we termed the CIR.

The CIR mediates direct and stable interaction with the CCR4–NOT. The fact that we could not delineate a single high-affinity linear motif in the CIR necessary and sufficient to mediate the interaction (data not shown) suggests that XRN1 binds to the CCR4–NOT via multiple motifs embedded within the CIR. Multiple purified, recombinant modules and subcomplexes of CCR4–NOT bind the CIR in pulldown assays, which is also consistent with an extended binding interface. The C-terminal NOT module appears to be the most prominent of the CCR4–NOT modules to interact with the CIR. This module acts as a ‘hub’ for protein-protein interactions with several mRNA specificity factors such as the *Dm* and vertebrate Nanos, the *Dm* Bicaudal C and the human transcription factor ERG (73–75). We also show that the CIR directly interacts with the CAF1 exonuclease but not with the nuclease domain of CCR4, and this is consistent with our observations that the CIR blocked CAF1- but not CCR4-mediated deadenylase activity *in vitro*. Direct binding to the CAF1 exonuclease—supported by additional stabilizing interactions with other parts of the CCR4–NOT—is likely crucial for the repressive effect of XRN1 on the CCR4–NOT-mediated mRNA deadenylation *in vivo* and *in vitro*.

The C-terminal structured region of PatL1 can directly compete with the CCR4–NOT complex for binding to the CIR and the interactions are mutually exclusive, which suggests that the XRN1-mediated inhibition of deadenylation may be regulated by PatL1 (Figure 6). Titrating recombinant PatL1-C protein *in vitro* appears to relieve the CIR-mediated inhibition of the CCR4–NOT deadenylase activity on synthetic RNA substrates. Interestingly, the CIR can interact with RNA directly *in vitro* but this interaction is not affected by the addition of PatL1-C. This is consistent with a model in which the XRN1 CIR represses CCR4–NOT-mediated deadenylation via a direct protein–protein interaction rather than by competing for the substrate and that PatL1-C relieves this repression by displacing the CCR4–NOT from XRN1 by direct competition for a mutually exclusive binding region within the CIR.

Deadenylation and decapping are tightly coupled events in the 5′–3′ decay pathway and PatL1 was proposed to be a key mediator of this coupling (40). We speculate that XRN1 may play a previously unappreciated role in regulating or coordinating molecular events at the opposite ends of a transcript. One possible scenario where this may be relevant is through early recruitment of XRN1 to the CCR4–NOT to impose a constraint to prevent premature deadenylation thus eliciting temporal control to dictate a certain sequence of events. Once PatL1 and the necessary decapping factors are recruited and correctly assembled to activate the DCP2 decapping enzyme, the deadenylation repression is relieved and degradation can proceed—very rapidly—with all required components of the decay machinery already in place and poised on the target mRNA.

The deadenylated and decapped tethered mRNA reporter is stabilized in XRN1-null cells and a transcriptome-wide analysis provided evidence for an accumulation of bulk mRNA decay intermediates that are not translated. In complementation assays in the XRN1-null cells, the catalytic domain of XRN1 alone was insufficient to rescue the mRNA decay defect but the C-terminal EDC4-binding motif and the CIR were both required although the extent differed. Finer dissection of the contribution of the low-complexity C-terminal region of XRN1 toward coordination of decay using complementation analysis revealed two intriguing observations. First, interfering with the EDC4 binding to the XRN1 stabilized incompletely deadenylated decay intermediates rather than a fully deadenylated mRNA reporter. Second, an XRN1 fragment that includes the CIR and the EDC4 binding motif does not repress deadenylation, unlike the CIR on its own. This suggests that the decapping factor EDC4—via direct binding to the XRN1—relieves the CIR-mediated repression of the CCR4–NOT deadenylase.

Taken together, our studies are consistent with a model in which XRN1 binding to upstream factors during mRNA decay is necessary for efficient recruitment of the exonuclease to its target mRNA and possibly to enable efficient and timely decapping. The regulatory role of XRN1 in mRNA decay might indeed alternate depending on the availability of binding partners—repressing deadenylation when bound to CCR4–NOT or stimulating decapping by interaction with PatL1 and EDC4 (Figure 6). Further biochemical and structural studies with purified recombinant factors supported by functional validation in cells will be necessary to elucidate the intricate network of interactions coordinated by XRN1 in the regulation of bulk and targeted 5′–3′ mRNA decay.

## DATA AVAILABILITY

Raw sequencing reads as well as processed data files corresponding to read counts and normalized abundance measurements generated in this study were deposited in the GEO under the accession number of GSE132725.

## Supplementary Material

gkz633_Supplemental_File
