# Supplementary material for: A low-complexity region in human XRN1 directly recruits deadenylation and decapping factors in 5′–3′ messenger RNA decay
Source: Nucleic Acids Res. 2019 Jul 24;47(17):9282–95. doi: 10.1093/nar/gkz633 (PMC6753473; doi:10.1093/nar/gkz633)
Supplement: gkz633_Supplemental_File [file gkz633_supplemental_file.pdf]

SUPPLEMENTARY INFORMATION

A low-complexity region in human XRN1 directly recruits deadenylation and decapping factors in 5′–3′ messenger RNA decay

Chung-Te Chang, Sowndarya Muthukumar, Ramona Weber, Yevgen Levdansky, Ying Chen, Dipankar Bhandari, Catia Igreja, Lara Wohlbold, Eugene Valkov, Elisa Izaurralde

Department of Biochemistry, Max Planck Institute for Developmental Biology, Max-Planck-Ring 5, 72076 Tübingen, Germany

Supplementary Table 1. Constructs and mutants used in this study.

Name	Fragments / mutations	Plasmid
GFP-MBP	-	pT7-EGFP-C1-MBP
GFP-*Hs*DCP2*	-	pT7-EGFP-C1-HsDCP2_E148Q
GFP-*Hs*XRN1	-	pT7-EGFP-C1-HsXRN1
	Cat	pT7-EGFP-C1-HsXRN1_1-1173
	CIR	pT7-EGFP-C1-HsXRN1_1174-1649
	EDC4-BM	pT7-EGFP-C1-HsXRN1_1650-1706
GFP-CAF1	-	pT7-EGFP-C1-HsNOT7
	CAF1*	pT7-EGFP-C1-HsNOT7_DExAA
GFP-POP2*	-	pT7-EGFP-C1-POP2_DExAA
HA-*Hs*PatL1	-	pT7-LN-HA-HsPatL1
	N+P	pT7-LN-HA-HsPatL1_1-398
	M+C	pT7-LN-HA-HsPatL1_399-770
GFP-*Hs*PatL1	-	pT7-EGFP-C1-HsPatL1
	ΔC	pT7-EGFP-C1-HsPatL1_1-517
β-globin-GAP	-	pcDNA3.1(+)-bglobin-GAP
β-globin-6xMS2bs	-	pcDNA3.1(+)-bglobin-6xMS2bs
MS2-HA	-	pcDNA3.1(+)-MS2-HA
MS2-HA-*Hs*SMG7	-	pcDNA3.1(+)-MS2-HA-HsSMG7
MS2-HA-NOT1	-	pT7-MS2-HA-HsNOT1
MS2-HA-Nanos1	-	pcDNA3.1(+)-MS2-HA-HsNanos1
HA-MBP	-	pCIneo-LN-HA-MBP
GFP-MBP	-	pT7-EGFP-C1-MBP
MBP-Strep	-	pnEK-NvHM-Strep-MBP
MBP-CIR-Strep	-	pETM-41P-HsXRN1_1174-1649-Strep
MBP-PNRC2-Strep	-	pnYC-pM-HsPNRC2-Strep
CCR4-NOT complex	-	pACEBac-NOT1-CAF40-NOT2-NOT3
		pMCSG19c-CCR4a
		pET28b-SUMO-CAF1
		pnYC-pM-NOT10_25-707
		pnEA-NOT11_257-498-vH
NOT10/11 module	-	pnYC-NOT1_1-682
		pnEA-NOT10_25-707-NOT11_257-498-vH
Catalytic module	-	pnYC-pM-NOT1_1093-1317
		pnEA-HsNOT7-NpM-HsNOT6
CAF40 module	-	pnYC-pM-NOT1_1351-1588
		pnEA-pH-CAF40_19-285
NOT module	-	pnYC-pM-NOT1_1833-2361
		pnEA-pH-NOT3_607-748-pM-NOT2_350-540
$CCR4^{cat}$	-	pnEA-NpM-NOT6_159-557
CAF1	-	pnYC-NvM-NOT7
*Hs*PatL1-C	-	pRSFDuetP-HsPatL1_517-770

Supplementary Table 2. Antibodies used in this study.

Name	Source	Catalog #	Dilution
anti-GFP (IP)	in house	—	1:200
anti-GFP (WB)	Roche	11 814 460 001	1:2,000
anti-NOT1	in house	—	1:1,000
anti-NOT2	Bethyl	A302-562A	1:1,000
anti-NOT3	abcam	ab55681	1:2,000
anti-CCR4	abcam	ab86209	1:1,000
anti-CAF1	Abnova	H00029883-M01	1:1,000
anti-*Hs*PAN2	Proteintech	16427-1-AP	1:500
anti-*Hs*PAN3	in house	—	1:1,000
anti-HA-HRP	Roche	12 013 819 001	1:5,000
anti-mouse-HRP	GE Healthcare	NA931V	1:10,000
anti-XRN1	Bethyl	A300-443A	1:1000

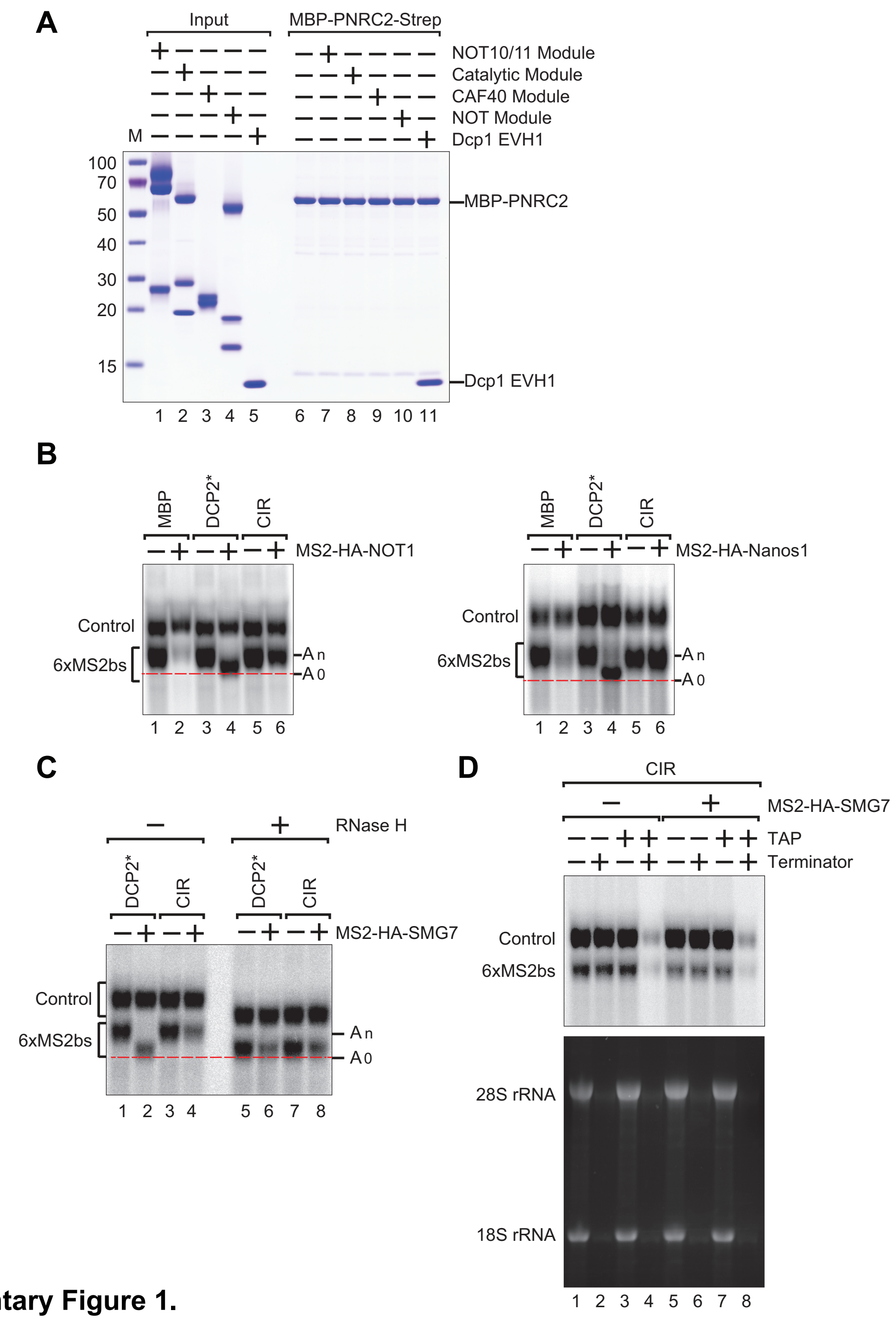

Supplementary Figure 1.

- **(A)** A streptavidin pulldown assay with MBP/Strep-tagged PNRC2 and purified recombinant CCR4-NOT modules, performed as described in Figure 1F. Recombinant Dcp1 served as a positive control for interaction with PNRC2.
- **(B)** Tethering assays with the β-globin-6xMS2bs reporter essentially performed as described in Figure 2A, except that MS2-HA-SMG7 was substituted by MS2-HA-NOT1 (left panel) or MS2-HA-Nanos1 (right panel).
- **(C)** The RNA samples isolated from cells expressing DCP2* or CIR (shown in Figure 2A) were treated with oligo(dT)$_{15}$ +/- RNase H and analyzed by northern blotting.
- **(D)** The RNA samples from Figure 2A were incubated with Terminator 5′-phosphate-dependent exonuclease in the absence or presence of tobacco acid pyrophosphatase (TAP) and analyzed by northern blotting. 28S and 18S ribosomal RNA served as uncapped control.

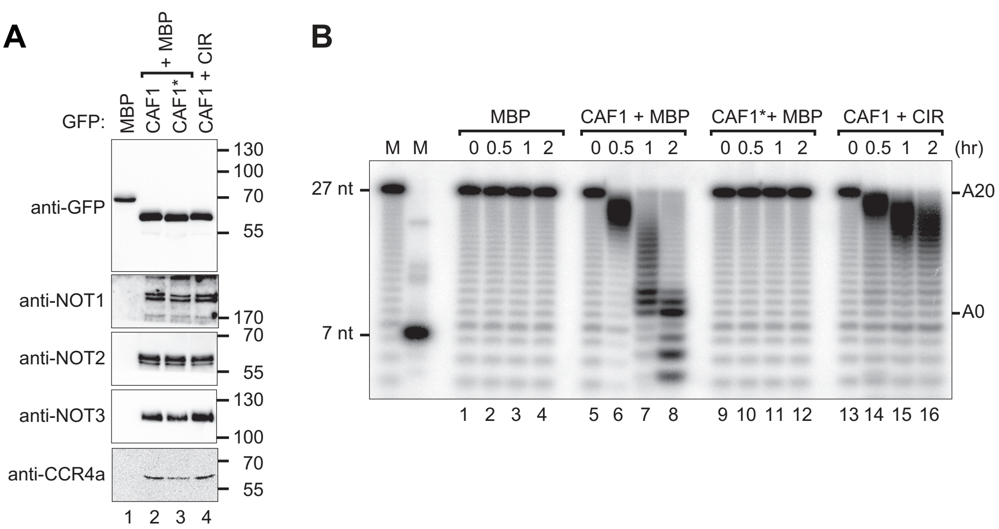

Supplementary Figure 2.

(A) GFP-tagged MBP or CAF1 (wild-type or catalytic mutant CAF1*, DExAA) were expressed in human HEK293T cells. GFP-tagged proteins were immunoprecipitated using anti-GFP antibodies. Precipitates were incubated with purified MBP or MBP-tagged CIR on beads before washing steps.

(B) The cleared immunoprecipitates from (A) were tested for deadenylase activity by incubation with a 27 nt 5′-^{32}P-labeled RNA substrate containing a poly(A) stretch. The reaction was terminated at the indicated time points by the addition of 2x RNA loading buffer.

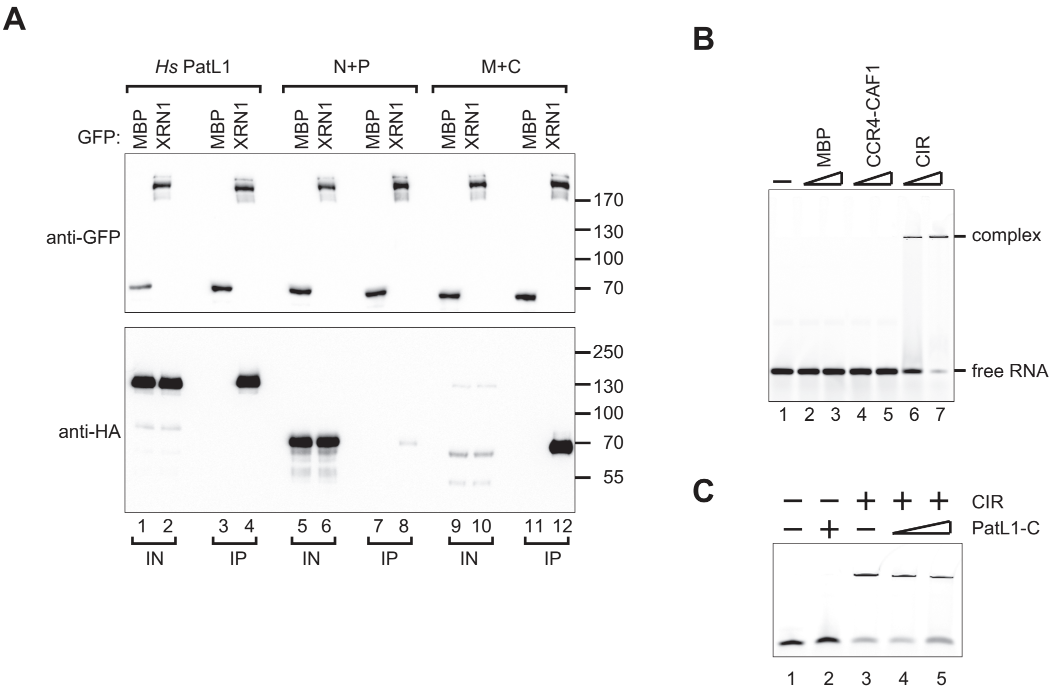

Supplementary Figure 3.

- **(A)** Immunoprecipitation assay showing the interaction of GFP-tagged XRN1 with HA-tagged PatL1 (full length or indicated fragments) in human HEK293T cells. Inputs (IN; 20% for GFP-tagged proteins and 10% for HA-tagged proteins) and bound fractions (IP; 20%) were analyzed by western blotting. GFP-tagged MBP served as a negative control.
- **(B)** Electrophoretic mobility shift assay (EMSA). Recombinant CCR4/CAF1 heterodimer or CIR were incubated with 27 nt 5′-6-FAM-labeled RNA substrate at 25 °C for 5 min. MBP served as a control. RNA-protein complex formation was analyzed by electrophoresis on a 10 % nondenaturing polyacrylamide gel.
- **(C)** EMSA essentially performed as in (B) with the indicated protein combinations. No RNA binding by PatL1-C alone was detected at the highest concentration (4 μM) used in this assay (lane 2).

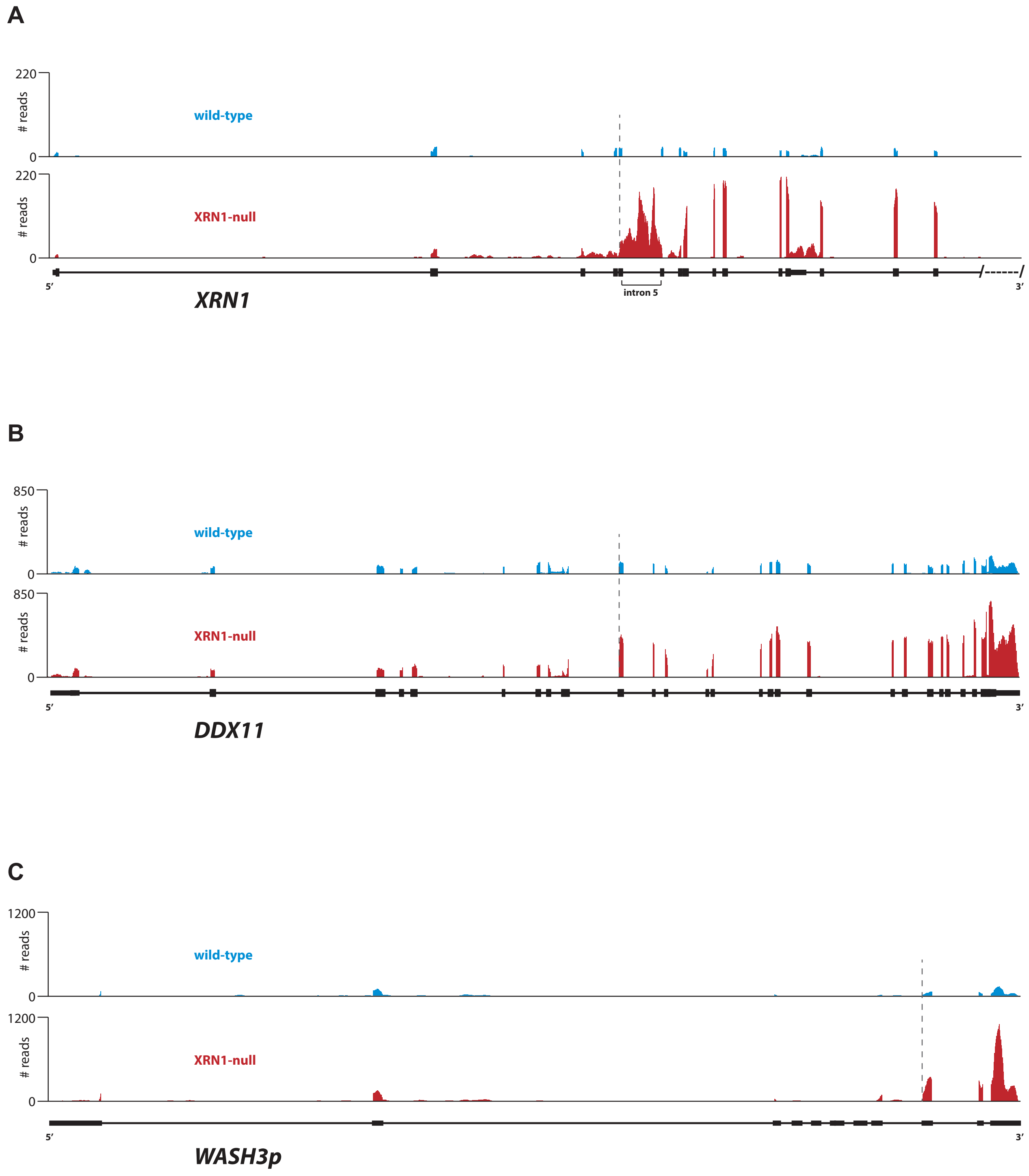

Supplementary Figure 4.

Mapped read coverage from RNA-Seq data for *XRN1* (A), *DDX11* (B), and *WASH3p* (C) mRNA in HEK293T WT and XRN1-null cells. The high read coverage of the XRN1 intron 5 in the XRN1-null cells is consistent with disrupted splicing as a consequence of the CRISPR/Cas9-mediated targeting of a sequence in exon 6. Inefficient splicing typically elicits nuclear retention and/or triggers NMD; however, defective exonucleolytic mRNA decay in the XRN1-null cells results in the accumulation of non-translatable *XRN1* mRNA decay intermediates. The abrupt increase in read coverage indicated by vertical dashed lines is consistent with an endonucleolytic cleavage of the identified transcripts. As a consequence of such cleavage, uncapped and thus translationally-incompetent mRNA fragments accumulate in the XRN1-null cells.

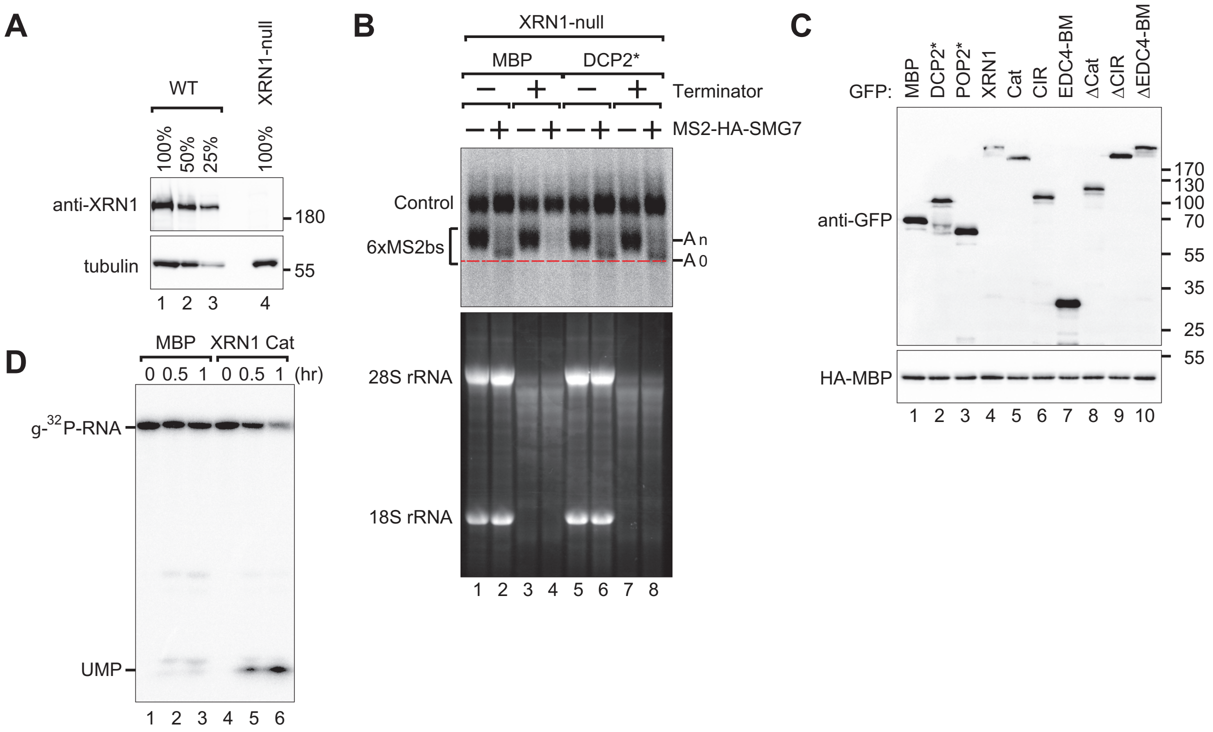

Supplementary Figure 5.

- **(A)** Detected XRN1 protein levels in HEK293T wild-type (WT) or XRN1-null cells.
- **(B)** Terminator 5′-phosphate exonuclease assay to test whether the reporter mRNA decay intermediate observed after SMG7 tethering in the XRN1-null cells is capped. RNA samples from Figure 5C, lanes 7-10 were incubated with or without Terminator 5′-phosphate-dependent exonuclease and analyzed by northern blotting. 28S and 18S ribosomal RNA served as uncapped control.
- **(C)** A western blot demonstrating the expression of the GFP-tagged proteins used in Figure 5E, F. HA-MBP served as a loading control.
- **(D)** GFP-tagged MBP or XRN1 catalytic domain (Cat) was expressed in human HEK293T cells and immunoprecipitated using anti-GFP antibodies. The immunoprecipitates were tested for 5′–3′ exoribonuclease activity with a synthetic 5’-labeled RNA substrate.
